# Foodborne illness outbreaks attributed to chemical hazards in the United States, 2011–2025

**DOI:** 10.3389/ftox.2026.1856871

**Published:** 2026-05-26

**Authors:** Brett Weed, Lauren Yeung, Omari Bandele, Cary Chen Parker

**Affiliations:** Human Foods Program, US Food and Drug Administration, College Park, MD, United States

**Keywords:** chemical adulteration, FDA, food safety, foodborne illness, toxins and toxicants in food

## Abstract

The Coordinated Outbreak Response and Evaluation (CORE) Network within the U.S. Food and Drug Administration (FDA) manages the investigation of multistate outbreaks of foodborne illness. From 2011 to 2025, CORE evaluated 1,270 foodborne illness incidents, of which 127 (10%) were attributable to a chemical exposure. Seafood toxins, from both shellfish and finfish, represented the majority of these incidents, followed by illicit or pharmaceutical drugs, toxic elements, and botanical toxins. Several case studies are presented which represent an array of classes of chemical hazards and distinct root causes of contamination. The detection and investigation of chemical foodborne incidents present additional complexities as compared to microbial outbreaks. These challenges include a lack of established surveillance and reporting systems for chemical incidents, difficulties in demonstrating a connection between illness presentation and a specific exposure to a given food product, and the heterogeneity of contamination. Additionally, many outbreaks require development and validation of new analytical methods for a novel food-contaminant pair to generate laboratory results sufficient to support regulatory action. FDA is committed to detecting, mitigating, and preventing chemical hazards in foods, including targeted public health surveillance programs, laboratory method development, and partnerships with other public health and food safety agencies to ensure a safe food supply for U.S. consumers.

## Introduction

Foodborne illness can be caused by biological, chemical, or physical hazards (FDA, 2024b). While extensive research has been dedicated to estimating the burden attributable to microbial pathogens, estimates of the total burden of illness due to chemical hazards are not well-defined (Choudhury et al., 2022). Foodborne illnesses from just four toxic metals (arsenic, cadmium, lead, and methylmercury) were estimated at one million cases and 56,000 deaths globally in 2015 (Gibb et al., 2019).

Investigations of foodborne exposure to chemical substances are complex and often a causative agent cannot be definitively identified (Ruff et al., 2021). Here, we summarize the incidents in the United States evaluated by the FDA Coordinated Outbreak Response and Evaluation (CORE) Network from its inception in 2011 to the present where a chemical hazard was identified as the confirmed or likely cause of foodborne illness. In this manuscript, “chemical hazard” refers to the causative chemical agent or contaminant, whereas “chemical incident” refers to a foodborne illness event or outbreak investigation attributed to such a hazard.

## Evaluating incidents attributable to chemical exposures

### The foodborne illness investigation process

Foodborne illness outbreak investigations involve coordinated efforts among federal, state, and local public health and regulatory agencies to identify outbreak sources and prevent additional illnesses (FDA, 2024a; CDC, 2025a). Investigators collect and analyze three types of data to determine the common food consumed by ill people. First, epidemiologic data are gathered as state and local partners work with the Centers for Disease Control and Prevention (CDC) to identify outbreaks and common foods consumed by ill individuals through public health surveillance (CDC, 2025b). Second, traceback data are collected when state and local authorities collaborate with U.S. Food and Drug Administration (FDA) to examine the food supply chain and determine the origin of suspected foods identified from epidemiologic evidence (Irvin et al., 2021; Seelman et al., 2021; Viazis et al., 2022). Finally, laboratory data are obtained through product and environmental sampling. These three types of data are evaluated holistically to confirm the contaminated food, with confirmation requiring support from at least two of the three categories. While outbreaks due to microbial hazards are supported by well-established surveillance systems and laboratory protocols, chemical incidents often rely on less formal or syndromic surveillance and often present obstacles to rapid laboratory identification of chemical hazards.

CORE serves as FDA’s coordination focal point for outbreak investigations in foods and dietary supplements. CORE comprises eight specialized teams, each with distinct responsibilities in the outbreak response process (FDA, 2024a). These teams evaluate emerging outbreaks and disease surveillance trends in collaboration with CDC, FDA field offices, and state/local agencies. They coordinate investigations, inspections, sampling, and product distribution tracing to control and stop outbreaks. The teams also monitor investigations and issue public warnings when there is ongoing risk with actionable steps to reduce illness. Following outbreak responses, CORE conducts analyses to recommend preventative measures for food safety activities.

### Data collection and analysis

The data for this analysis were extracted from the CORE database of foodborne illness incidents. The CORE database includes detailed information on foodborne outbreaks that were evaluated and/or investigated by the FDA from the creation of CORE in 2011 to the present. While this data does not represent all outbreaks associated with FDA-regulated human foods, it does represent outbreaks with FDA involvement. The database includes information on the number of illnesses, the food vehicle and implicated hazard.

All outbreaks not associated with a microbial pathogen were reviewed for inclusion. Incidents were included and classified based on the confirmed or suspected chemical hazard associated with the illnesses. Incidents were classified by broad category of exposure: seafood; pharmaceutical or illicit drugs; toxic elements; natural toxins; dietary supplement ingredients; industrial chemicals; food additives; cosmetics; or unknown/undetermined. Seafood toxins were further classified into scombrotoxin, ciguatoxin, tetrodotoxin, shellfish toxins, or other. Diagrams were generated using SankeyMATIC software.

### Types of incidents attributed to chemical hazards, 2011–2025

**FIGURE 1:**
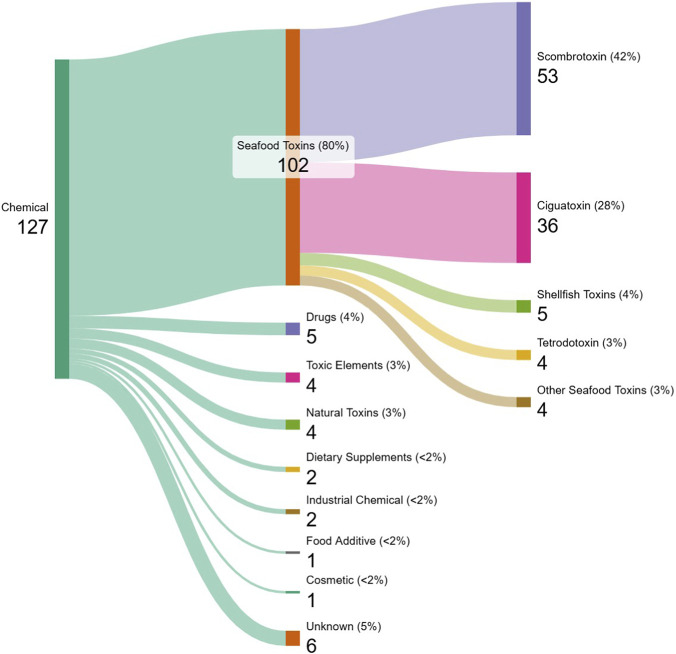
Source attribution of chemical foodborne illness investigated by the FDA CORE network, 2011–2025 (frequency, percentage).

From June 2011 through April 2025, CORE evaluated a total of 1,270 foodborne incidents. A total of 127 incidents (127/1,270; 10%) were classified as a suspected or confirmed chemical hazard as the cause of illness across twelve exposure categories (Figure 1), with the remaining 90% attributable to microbial hazards. Most chemical incidents were attributable to toxins associated with fishery products (102/127; 80%), including scombrotoxin, ciguatoxin, tetrodotoxin, and shellfish toxins such as brevetoxins or domoic acid. The remaining incidents were attributed to adulteration with pharmaceutical and/or illicit drug substances (5), toxic elements (4), natural toxins (4), dietary supplement ingredients (2), industrial chemicals (2), food additives (1), and cosmetic ingredients (1). In six cases, the precise chemical constituent or toxic exposure could not be identified. Several of the drug substance incidents involved contamination of dietary supplements with unsafe or undeclared drugs. The cases categorized as dietary supplement ingredients are incidents where the toxic effects were attributable to the declared dietary supplement component itself.

### Case studies in chemical hazard-attributable outbreaks

The following case studies highlight the varied food categories and chemical hazard types identified during CORE investigations.

#### Seafood toxin outbreaks

An estimated 3% of foodborne illnesses in the United States are attributable to fish, with over 90% of fishery product outbreaks due to either scombrotoxin (55%) or ciguatoxin (36%) (Barrett et al., 2017). CORE outbreak data mirror these published estimates, with 80% of seafood outbreaks linked to toxins.

In 2018, CORE modified its incident evaluation process to exclude seafood toxin outbreaks which were isolated to a single exposure or retail point of service. Initiation of outbreak investigations for seafood toxins are now reserved for those incidents where opportunities for FDA public health interventions exist, such as a large 2019 scombrotoxin outbreak linked to deficiencies in seafood handling at harvest in Vietnam (Pereira et al., 2021). As a result, the proportion of seafood toxin outbreaks among incidents investigated by CORE is expected to decline over time, though still represents a true and persistent hazard.

#### Niacin overenrichment, 2013 (food additive)

Outbreaks due to niacin overenrichment of grain products have been previously noted in the literature (Bartlett et al., 1982; Hudson and Vogt, 1985). In December 2013, CORE was notified of illnesses in schools and institutional settings in three states (TX, IL, ND). Epidemiologic investigation identified illnesses occurring shortly after consumption of an infused rice product. Symptoms included redness, flushing, and rashes, which are consistent with adverse effects previously reported from excess niacin exposure (Ginsburg, 2019). FDA laboratory analysis determined the rice products had niacin levels far in excess of the amounts indicated on the product labels. A total of 81 cases were identified in the outbreak. Due to the investigative findings, the manufacturer recalled the overenriched products (Sun, 2014).

#### Adulteration of applesauce with lead chromate, 2023 (toxic element)

In October 2023, public health officials in North Carolina detected several children with high blood lead levels who had each eaten the same cinnamon-flavored applesauce product. Initial testing found extremely high levels of lead in the product (Troeschel et al., 2025). In response, an international investigation led by FDA and CDC was launched to determine the source and prevent ongoing contamination.

The investigation determined that cinnamon ground in Ecuador was the source with levels reaching as much as 6.43 ppm in the applesauce, which is more than 600-fold higher than the FDA action level for infants and toddlers (FDA, 2025a). Novel laboratory methods developed at FDA’s National Forensic Chemistry Center (NFCC) determined the particles in the ground cinnamon to be lead chromate, which has been previously implicated as an economic adulterant in spices to improve color and weight (Cowell et al., 2017; Forsyth et al., 2019).

#### Consumption of raw morel mushrooms, 2023 (natural toxin)

In 2023, FDA was notified by Montana health authorities of a cluster of illnesses associated with consumption of sushi rolls containing raw or undercooked morel mushrooms. Morels are recognized as being capable of causing neurological and gastrointestinal symptoms if eaten raw (Goldfrank, 2019) and a proposed taxonomy of mushroom toxicity published in 2019 includes *morel neurological syndrome* as a distinct category (White et al., 2019).

Fifty-one cases of illness were identified, including three hospitalizations and two deaths (Demorest et al., 2024). Symptoms were primarily gastrointestinal in nature, including vomiting, diarrhea, fatigue, loss of appetite, and abdominal pain. FDA laboratory analyses of mushroom samples were negative for bacterial toxins, heavy metals, hydrazine, pesticides, and common poisons. DNA analysis was performed and confirmed the mushrooms were properly identified as true morels (*Morchella sextelata*) (Tesfai et al., 2025). Notably, mushrooms from the same source/lot were supplied elsewhere, each of which thoroughly cooked the mushrooms and reported no adverse events.

## Discussion

While chemical outbreaks represent a relatively small proportion of incidents investigated by CORE, these cases can have severe health outcomes. Even these rare events warrant attention due to the unique challenges presented in investigating them.

### Challenges during chemical incidents

In addition to identifying the source of foodborne illness and implementing control measures to mitigate risks to public health, FDA outbreak investigations also seek to identify the root causes of food adulteration to reduce or prevent future outbreaks. The causes of contamination are highly variable, including failures in food safety practices throughout the supply chain, economic fraud, or intentional adulteration. Examples of root causes identified in select investigations are summarized in Table 1.

**TABLE 1:** Example causes of chemical hazards in foods and example incidents in foodborne illness incidents investigated by the US Food and Drug Administration, 2011–2025.

Root cause	Example hazard-food pair incidents [outbreak identifier]^
Inadequate Food Safety Practices (Growing)	Organophosphate Pesticide Residue-Spinach Leaves [#1264]
Inadequate Food Safety Practices (Formulation)	Tara Root Flour-Lentil and Leek Crumbles [#1076]
Inadequate Food Safety Practices (Processing, Production, and Holding)	Niacin Overenrichment-Infused Rice [#339]
Economically-Motivated Adulteration	Non-Edible Metallic Luster Dust in Baked Goods (Viveiros et al., 2021) Melamine Monomer in Milk Powders (Gossner et al., 2009)
Intentional Acts	Lead Chromate-Apple Cinnamon Purees [#1198] Psychoactive Substances and Pharmaceutical Agents in Candy Products [#1233]

^Outbreak Identifier as identified in Supplementary Table S1 (if applicable).

### Complexities of epidemiological and laboratory data collection

Chemical incidents often are detected by novel data streams outside of routine foodborne illness surveillance, which often lack full clinical and product details needed for investigation (Bazaco et al., 2025). These outbreak investigations often require additional resources to collect and investigate cases, in addition to definitive identification of the toxic agent(s) implicated.

Laboratory results are a crucial piece of evidence used to support public health and regulatory interventions intended to mitigate or prevent foodborne illness. Chemical outbreaks are more likely to involve complex mixtures or unknown contaminants requiring multi-faceted testing to identify a presumptive cause. Regulatory laboratory assays are often validated for specific analyte-matrix pairs, restricting the applicability of existing test methods to specific foods or classes of food products (e.g., aflatoxin in corn). Chemical outbreaks are more likely to involve novel compounds lacking validated methods in foods, or the existing methods are qualitative screens, whereas typically a validated quantitative method is needed to support regulatory action. In these situations, new regulatory laboratory methods must be developed and validated in real-time.

### Source attribution

Chemical outbreaks present unique challenges that can delay identification of both the chemical agent and contaminated food item (Onyeaka et al., 2024). Unlike microbial outbreaks, where established diagnostics, genetic subtyping, and surveillance systems support more timely source identification, chemical incidents lack analogous tools for source tracking. A major challenge is the non-specific and variable clinical presentation of acute chemical intoxication. Symptoms such as nausea, vomiting, abdominal pain, and/or dizziness frequently overlap with those caused by microbial agents, often leading to initial misclassification (Schier et al., 2006). The onset of symptoms may be delayed or subclinical, and the severity can vary based on individual susceptibility, age, health status, or co-exposures, which can hinder epidemiologic recognition (Schier et al., 2006; Onyeaka et al., 2024).

Contaminant identification is further hindered by limited access to validated analytical methods, appropriate reference standards, and specialized instrumentation capable of detecting a wide range of contaminants in complex food matrices (National Institute of Standards and Technology, 2020; Ahuja et al., 2023; Li et al., 2024). Public health laboratories may lack the capacity to detect novel or unexpected chemicals, particularly those not included in routine screening panels for known chemical contaminants (e.g., metals, mycotoxins, histamine, *etc.*) In many cases, the food may no longer be available by the time symptoms are recognized or the chemical may have degraded before testing can be conducted.

The chemical complexity of food, including mixtures of proteins, lipids, carbohydrates, and additives, adds another layer of analytical challenges. Even when the causative chemical is identified, tracing it to a specific source is typically not straightforward (Ahuja et al., 2023; Falsafi et al., 2025). Contaminants may enter food through naturally occurring toxins in raw ingredients, environmental contamination, processing, packaging materials, or intentionally added for economic gain or malicious purposes (Rather et al., 2017). These varied exposure pathways, combined with the globalized nature of modern food supply chains, make traceback investigations for foodborne chemical outbreaks particularly challenging and potentially hinders timely public health intervention and response (Aybar Espinoza et al., 2026).

### Heterogenous distribution of chemical contaminations in food products

Public health responses to foodborne chemical outbreaks are complicated in part by heterogenous distribution of the chemical contaminant in food products. These challenges hinder the identification of the causative agent, exposure assessments, remediation efforts, and effective risk communication.

Chemical contaminants are frequently distributed heterogeneously across affected batches or even within a single food item. This heterogeneity can result from inconsistent mixing during processing, variations in chemical stability, differential migration from packaging under varying storage conditions, or localized contamination incidents (Cullen and O'Donnell, 2009; Biant et al., 2025). As one example, melamine added fraudulently to dairy products (e.g., milk and infant formula) to falsely elevate protein measurements in China in 2008 was not evenly distributed in all products with levels varying significantly among production batches (Ingelfinger, 2008; Gossner et al., 2009). This inconsistency stemmed from irregular adulteration practices by suppliers, including the use of melamine of differing purity and non-uniform mixing. Consequently, certain lots of infant formula caused severe renal toxicity (e.g., kidney stones and renal failure) in infants while others with lower levels of melamine were not linked to any toxic effects (Zhu et al., 2009). Such variability hindered response efforts because it was difficult to quickly identify which products were highly contaminated (EFSA Panel on Contaminants, 2010). In general, the heterogenous distribution of chemical hazards, regardless of the intentional or unintentional nature of the contamination, requires extensive sampling to detect “hot spots” and to avoid false negatives or underestimation of exposure (Biant et al., 2025). Thus, heterogenous contaminant distribution prevents reliable exposure assessments and timely identification of the causative agent in foodborne chemical outbreaks.

### Interaction between contaminants and food matrices

Another major challenge is that chemical contaminants rarely exist in isolation. Interactions between the contaminant and other constituents within the food matrix can alter their toxicological effects. This can potentially alter how much contaminant is absorbed in the gut, how it is distributed and metabolized in the body, and ultimately its toxicological effects (More et al., 2019). Foods contain complex mixtures of nutrients, additives, and possibly other contaminants, any of which may interact with a given toxicant. These interactions can produce additive, synergistic, or antagonistic effects, complicating predictions which are typically based on the toxicological profile of the individual contaminant alone (Hernández et al., 2017). Again in the example above, the combination of melamine and cyanuric acid from melamine production caused renal toxicity mediated by the formation of melamine-cyanurate crystals, which significantly exceeded the predicted effects based on evaluations of the chemicals individually (i.e., synergistic effect) (Gossner et al., 2009; World Health Organization, 2009). Chemical reactions within the food matrix can transform contaminants into new compounds that display different toxicity. For example, nitrite preservatives used in cured meats can react with amines in the meat to form N-nitrosamines, classified as carcinogens, that are considered more hazardous than nitrite alone (Tricker and Preussmann, 1991; Cassens, 1997; International Agency for Research on Cancer, 2010). Also, lipid-rich foods can alter the bioavailability and toxicokinetic profile of lipophilic contaminants such as dioxins and polychlorinated biphenyls (PCBs) (EFSA Panel on Contaminants in the Food Chain (CONTAM), 2018). Such chemical transformations and interactions add complexities to exposure and risk assessments, which can hamper outbreak response efforts (Domingo and Bocio, 2007; More et al., 2019). Thus, toxicological evaluations based on a single chemical in isolation may not accurately predict the real-world hazard when the chemical is ingested as part of a complex food matrix containing multiple diverse substances.

### Mitigation efforts and FDA’s role in protecting the food supply

Following foodborne illness outbreaks, FDA considers whether surveillance sampling would be an appropriate mitigation and monitoring strategy or is necessary to adequately assess baseline exposure and risk. Factors considered include whether adequate data already exist to understand the source and prevalence of the hazard, likelihood of the incident occurring again, FDA jurisdiction over the product and/or ingredients suspected to be the cause, availability of validated laboratory methods for the product(s) to be tested, and availability of a compliance strategy to act on results. FDA may conduct this surveillance using internal resources, or partner with state food regulatory programs and laboratories who share a mutual interest in the topic.

An example of surveillance following a chemical event is the collection and testing of ground cinnamon following the 2023 cinnamon-flavored applesauce incident. Between December 2023 and March 2024, FDA collected 75 samples of food products containing ground cinnamon from discount retail stores and tested for lead and chromium. FDA also leveraged state partners through laboratory infrastructure grants to collect and analyze samples. Cumulatively, this surveillance has resulted in 20 recalls and seven FDA public health alerts through December 2025 (FDA, 2025b). In addition to ground cinnamon, the ongoing surveillance efforts have tested other spices, such as turmeric, cumin, and curry powder that may be susceptible to the same toxic element adulteration.

## Conclusion

While most foodborne outbreaks are attributable to microbiological contamination, approximately one in ten is caused by exposures to chemical hazards. From 2011 to 2025, FDA CORE evaluated 127 foodborne illness clusters confirmed or suspected of being due to chemical hazards. Eighty percent of incidents were seafood-associated toxins (including both shellfish and finfish), with the remaining 20% spread across illicit drugs, pharmaceuticals, industrial chemicals, food additives, dietary supplements, and cosmetic products.

Outbreaks attributed to chemical hazards are inherently more complex to identify and investigate for food safety regulators. Chemical incidents must often rely on non-traditional data sources for surveillance information and identification of an outbreak. Additionally, validated regulatory laboratory methods often do not exist for a specific chemical-food matrix prior to identification of the outbreak, requiring rapid method development during an active response. Despite these challenges, the FDA is committed to investigating, mitigating, and preventing illness and injury from chemical hazards in foods.

## Data Availability

The original contributions presented in the study are included in the article/Supplementary Material, further inquiries can be directed to the corresponding author.
