# Supplementary material for: Foodborne illness outbreaks attributed to chemical hazards in the United States, 2011–2025
Source: Front Toxicol. 2026 May 26;8:1856871. doi: 10.3389/ftox.2026.1856871 (PMC13245939; doi:10.3389/ftox.2026.1856871)
Supplement: Supplementary file 1 [file Table1.docx]

Supplementary Table 1. Chemical Incidents Investigated by the FDA Coordinated Outbreak Response and Evaluation (CORE) Network, 2011-2025

| **CORE Outbreak Identifier** | **Incident Name** | **Start Date** | **Category** | **Total Cases (if known)** |
| --- | --- | --- | --- | --- |
| 15 | Adverse Reaction/Black Henna/MN/Aug 2011 | 8/8/2011 | Cosmetic | 3 |
| 16 | Ciguatoxin/Black Grouper/NY/Aug 2011 | 8/8/2011 | Seafood Toxin | 2 |
| 17 | Scombrotoxin/Ahi Tuna/ID/Aug 2011 | 8/8/2011 | Seafood Toxin | 2 |
| 20 | Diarrheic Shellfish Poisoning/Mussels/WA/Aug 2011 | 8/9/2011 | Shellfish Toxin | Unknown |
| 26 | Scombrotoxin/Tuna (suspect)/OR/Aug 2011 | 8/26/2011 | Seafood Toxin | Unknown |
| 43 | Scombrotoxin/Tuna/OR/Sep 2011 | 9/29/2011 | Seafood Toxin | 3 |
| 61 | Scombrotoxin/Retort Tuna/OH/Nov 2011 | 11/9/2011 | Seafood Toxin | 2 |
| 66 | Ciguatoxin/Red Grouper (suspect)/NY/Nov 2011 | 11/21/2011 | Seafood Toxin | 3 |
| 68 | Scombrotoxin/Tuna (suspect)/MA/Nov 2011 | 11/25/2011 | Seafood Toxin | 5 |
| 82 | Shellfish Poisoning/Clams; Scallops (suspect)/ME/Jan 2012 | 1/26/2012 | Shellfish Toxin | Unknown |
| 85 | Scombrotoxin/Mahi Mahi (suspect)/MA/Feb 2012 | 2/9/2012 | Seafood Toxin | 2 |
| 172 | Rhabdomyolysis/Canned Mackerel (suspect)/TN/Sep 2012 | 9/22/2012 | Seafood Toxin | 42 |
| 189 | Scombrotoxin/Tuna/OH/Oct 2012 | 10/26/2012 | Seafood Toxin | 2 |
| 199 | Adverse Reaction/Dietary Supplement Beverage (suspect)/NJ/Dec 2012 | 12/5/2012 | Dietary Supplement | 1 |
| 209 | Scombrotoxin (suspect)/Sushi (suspect)/WI/Jan 2013 | 1/4/2013 | Seafood Toxin | Unknown |
| 217 | Scombrotoxin/Escolar/MT/Jan 2013 | 1/22/2013 | Seafood Toxin | 3 |
| 222 | Haff Disease/Carp (suspect)/NY/Feb 2013 | 2/4/2013 | Seafood Toxin | Unknown |
| 224 | Ciguatoxin/Barracuda/NY/Feb 2013 | 2/6/2013 | Seafood Toxin | Unknown |
| 236 | Scombrotoxin/Tuna/OR/Mar 2013 | 3/13/2013 | Seafood Toxin | Unknown |
| 241 | Ciguatoxin/Sea Bass (suspect)/MN/Mar 2013 | 3/27/2013 | Seafood Toxin | Unknown |
| 246 | Ciguatoxin/Escolar; Sea Bass (suspect)/MN/Mar 2013 | 3/28/2013 | Seafood Toxin | Unknown |
| 250 | Scombrotoxin/Tuna/RI/Apr 2013 | 4/17/2013 | Seafood Toxin | 1 |
| 259 | Scombrotoxin/Retort Tuna (suspect)/MN/May 2013 | 5/13/2013 | Seafood Toxin | 1 |
| 264 | Scombrotoxin/Ahi Tuna (suspect)/MD/May 2013 | 5/15/2013 | Seafood Toxin | 1 |
| 274 | Ciguatoxin/Fish/PR/May 2013 | 5/22/2013 | Seafood Toxin | Unknown |
| 272 | Scombrotoxin/Spicy Tuna/CT/May 2013 | 5/22/2013 | Seafood Toxin | Unknown |
| 273 | Scombrotoxin/Yellowfin Tuna (suspect)/FL/May 2013 | 5/22/2013 | Seafood Toxin | Unknown |
| 276 | Scombrotoxin/Round Scad (suspect)/CA/Jun 2013 | 6/4/2013 | Seafood Toxin | Unknown |
| 279 | Scombrotoxin/Canned Mackerel (suspect)/NY/Jun 2013 | 6/10/2013 | Seafood Toxin | Unknown |
| 282 | Ciguatoxin/Blue Marlin (suspect)/HI/Jun 2013 | 6/14/2013 | Seafood Toxin | Unknown |
| 288 | Scombrotoxin/Mahi Mahi (suspect)/RI/Jun 2013 | 6/24/2013 | Seafood Toxin | Unknown |
| 289 | Scombrotoxin/Tuna (suspect)/MD/Jun 2013 | 6/24/2013 | Seafood Toxin | Unknown |
| 298 | Amnesic Shellfish Poisoning/Mussels/PA/Jul 2013 | 7/6/2013 | Shellfish Toxin | 2 |
| 304 | Rhabdomyolysis/Buffalo fish (suspect)/MS/Jul 2013 | 7/20/2013 | Seafood Toxin | Unknown |
| 311 | Ciguatoxin/Amberjack (suspect)/FL/Aug 2013 | 8/8/2013 | Seafood Toxin | Unknown |
| 321 | Acute Liver Failure/Dietary Supplement (suspect)/Sep 2013 | 9/24/2013 | Pharmaceutical or Illicit Drug | 72 |
| 339 | Niacin Toxicity/Infused Rice/Dec 2013 | 12/4/2013 | Food Additive | 1 |
| 344 | Tetrodotoxin/Puffer Fish/VA/Jan 2014 | 1/13/2014 | Seafood Toxin | 8 |
| 349 | Rhabdomyolysis/Buffalo Fish (suspect)/IL/Feb 2014 | 2/4/2014 | Seafood Toxin | 4 |
| 359 | Ciguatoxin/Amberjack/GA/Mar 2014 | 3/18/2014 | Seafood Toxin | 7 |
| 360 | Skin rash/Breaded seasoned tilapia (suspect)/FL/Mar 2014 | 3/26/2014 | Unknown | Unknown |
| 368 | Ciguatoxin/Barracuda/NY/Apr 2014 | 4/9/2014 | Seafood Toxin | Unknown |
| 386 | Scombrotoxin/Yellowfin Tuna (suspect)/NY/Jun 2014 | 6/6/2014 | Seafood Toxin | Unknown |
| 392 | Tetrodotoxin/Puffer Fish (suspect)/MN/Jun 2014 | 6/18/2014 | Seafood Toxin | Unknown |
| 396 | Scombrotoxin/Bluefin Tuna (suspect)/NY/Jun 2014 | 6/27/2014 | Seafood Toxin | Unknown |
| 397 | Scombrotoxin/Unknown/WA/Jun 2014 | 6/27/2014 | Seafood Toxin | Unknown |
| 405 | Scombrotoxin/Mahi Mahi (suspect)/CA/Jul 2014 | 7/8/2014 | Seafood Toxin | Unknown |
| 407 | Ciguatoxin/Amberjack/FL/Jul 2014 | 7/15/2014 | Seafood Toxin | Unknown |
| 410 | Scombrotoxin/Yellowfin Tuna/NY/Jul 2014 | 7/21/2014 | Seafood Toxin | Unknown |
| 412 | Scombrotoxin/Swai Fish (suspect)/TN/Jul 2014 | 7/22/2014 | Seafood Toxin | Unknown |
| 411 | Heavy Metal Poisoning/Protein Powder (suspect)/WA/Jul 2014 | 7/22/2014 | Toxic Element | Unknown |
| 415 | Scombrotoxin/Mahi Mahi (suspect)/WA/Aug 2014 | 8/4/2014 | Seafood Toxin | Unknown |
| 416 | Scombrotoxin/Tuna/OH/Aug 2014 | 8/5/2014 | Seafood Toxin | Unknown |
| 424 | Scombrotoxin/Mahi Mahi (suspect)/FL/Aug 2014 | 8/19/2014 | Seafood Toxin | Unknown |
| 428 | Scombrotoxing/Retort Tuna (suspect)/NV/Sep 2014 | 9/3/2014 | Seafood Toxin | Unknown |
| 450 | Ciguatoxin/Grouper (suspect)/FL/Nov 2014 | 11/5/2014 | Seafood Toxin | Unknown |
| 452 | Scombrotoxin/Tuna/NY/Nov 2014 | 11/10/2014 | Seafood Toxin | Unknown |
| 462 | Scombrotoxin/Swordfish (suspect)/WA/Jan 2016 | 1/20/2015 | Seafood Toxin | Unknown |
| 463 | Adverse Reaction/Apple juice (suspect)/IL/Jan 2015 | 1/26/2015 | Unknown | Unknown |
| 469 | Scombrotoxin/Salmon/IA/Mar 2015 | 3/13/2015 | Seafood Toxin | 2 |
| 470 | Scombrotoxin/Salmon (suspect)/IA/Mar 2015 | 3/13/2015 | Seafood Toxin | Unknown |
| 483 | Scombrotoxin/Mahi Mahi (suspect)/WA/May 2015 | 5/19/2015 | Seafood Toxin | Unknown |
| 484 | Scombrotoxin/Tuna/TN/May 2015 | 5/19/2015 | Seafood Toxin | Unknown |
| 492 | Ciguatoxin/Barracuda (suspect)/MD/Jun 2015 | 6/29/2015 | Seafood Toxin | Unknown |
| 493 | Ciguatoxin/Black grouper/MD/Jun 2015 | 6/29/2015 | Seafood Toxin | Unknown |
| 495 | Tetrodotoxin/Puffer Fish/PA/Jul 2015 | 7/6/2015 | Seafood Toxin | Unknown |
| 502 | Scombrotoxin/Mahi Mahi (suspect)/MD/Aug 2015 | 8/17/2015 | Seafood Toxin | Unknown |
| 503 | Scombrotoxin/Tuna (suspect)/Aug 2015 | 8/20/2015 | Seafood Toxin | Unknown |
| 505 | Scombrotoxin/Tuna (suspect)/TN/Aug 2015 | 8/27/2015 | Seafood Toxin | Unknown |
| 516 | Ciguatoxin/Amberjack (suspect)/WI/Oct 2015 | 10/19/2015 | Seafood Toxin | Unknown |
| 547 | Elevated Liver enzymes/Dietary Supplement (suspect)/HI/Feb 2016 | 2/10/2016 | Dietary Supplement | Unknown |
| 548 | Amnesic Shellfish Poisoning/Unknown/MN/Feb 2016 | 2/12/2016 | Shellfish Toxin | Unknown |
| 549 | Scombrotoxin/Tuna (suspect)/MD/Feb 2016 | 2/18/2016 | Seafood Toxin | Unknown |
| 552 | Lead/Spices (suspect)/NC/Mar 2016 | 3/16/2016 | Toxic Element | Unknown |
| 567 | Scombrotoxin/Sardines (suspect)/WA/May 2016 | 5/16/2016 | Seafood Toxin | Unknown |
| 569 | Scombrotoxin/Ahi Tuna (suspect)/WA/May 2016 | 5/25/2016 | Seafood Toxin | Unknown |
| 574 | Scombrotoxin/Ahi Tuna/ND/Jun 2016 | 6/30/2016 | Seafood Toxin | Unknown |
| 588 | Lead/Dietary Supplement/IL/Aug 2016 | 8/24/2016 | Toxic Element | 5 |
| 593 | Ciguatoxin/Amberjack (suspect)/FL/Sep 2016 | 9/29/2016 | Seafood Toxin | Unknown |
| 594 | Tetrodotoxin/Unknown/VA/Oct 2016 | 10/19/2016 | Seafood Toxin | Unknown |
| 606 | Ciguatoxin/Barracuda (suspect)/FL/Dec 2016 | 12/6/2016 | Seafood Toxin | Unknown |
| 612 | Myalgia/Amberjack fish (suspect)/Brazil/Dec 2016 | 12/22/2016 | Unknown | Unknown |
| 618 | Ciguatoxin/Grouper or Mahi Mahi (suspect)/FL/Feb 2017 | 2/6/2017 | Seafood Toxin | Unknown |
| 623 | Scombrotoxin/Tuna (suspect)/IN/Feb 2017 | 2/27/2017 | Seafood Toxin | Unknown |
| 625 | Chemical (detergent)/Apple Juice (suspect)/PA/Mar 2017 | 3/7/2017 | Industrial Chemical | Unknown |
| 634 | Aconite poisonings/Herbal tea supplement (suspect)/CA/Mar2017 | 3/22/2017 | Natural Toxin | Unknown |
| 636 | Neurotoxic Shellfish Poisoning/Conch/FL/Mar 2017 | 3/28/2017 | Shellfish Toxin | Unknown |
| 645 | Ciguatoxin/Red snapper (suspect)/NJ/Apr 2017 | 4/28/2017 | Seafood Toxin | Unknown |
| 646 | Scombrotoxin/Tuna (suspect)/NY/Apr 2017 | 4/28/2017 | Seafood Toxin | Unknown |
| 668 | Ciguatoxin/Barracuda (suspect)/MA/Jun 2017 | 6/12/2017 | Seafood Toxin | Unknown |
| 682 | Ciguatoxin/Yellowtail Snapper/PR/Jul 2017 | 7/17/2017 | Seafood Toxin | Unknown |
| 694 | Ciguatera/barracuda/MD/Sep 2017 | 9/12/2017 | Seafood Toxin | Unknown |
| 699 | Scombrotoxin/Tuna (suspect)/KS/Sep 2017 | 9/28/2017 | Seafood Toxin | Unknown |
| 703 | Scombrotoxin/Mahi Mahi/UT/Oct 2017 | 10/12/2017 | Seafood Toxin | Unknown |
| 709 | Ciguatoxin/Barracuda/FL/Oct 2017 | 10/30/2017 | Seafood Toxin | Unknown |
| 710 | Scombrotoxin/Anchovies (suspect)/PA/Oct 2017 | 10/30/2017 | Seafood Toxin | Unknown |
| 716 | Adverse Reaction/Drug/Nov 2017 | 11/7/2017 | Pharmaceutical or Illicit Drug | 30 |
| 750 | Ciguatoxin/Snapper (suspect)/FL/Feb 2018 | 2/20/2018 | Seafood Toxin | Unknown |
| 751 | Acute Poisonings/Cannabidiol (CBD) Oil (suspect)/Feb 2018 | 2/22/2018 | Pharmaceutical or Illicit Drug | Unknown |
| 755 | Ciguatoxin/Giant Kingfish and Parrotfish (suspect)/HI/Mar 2018 | 3/13/2018 | Seafood Toxin | Unknown |
| 756 | Ciguatoxin/Amberjack (kahala) and Gray Snapper (uku) (suspect)/HI/Mar 2018 | 3/13/2018 | Seafood Toxin | Unknown |
| 761 | Severe Bleeding (Brodifacoum)/Synthetic Cannabinoid/Mar 2018 | 3/23/2018 | Pharmaceutical or Illicit Drug | Unknown |
| 764 | Ciguatoxin/Bigeye Emperor fish/HI/Apr 2018 | 4/4/2018 | Seafood Toxin | Unknown |
| 765 | Ciguatoxin/Barracuda/FL/Apr 2018 | 4/5/2018 | Seafood Toxin | Unknown |
| 772 | Scombrotoxin/Tuna (suspect)/WA/Apr 2018 | 4/24/2018 | Seafood Toxin | Unknown |
| 774 | Ciguatoxin/Wild Dover Sole (suspect)/CA/Apr 2018 | 4/30/2018 | Seafood Toxin | Unknown |
| 781 | Ciguatoxin/Barracuda/FL/May 2018 | 5/22/2018 | Seafood Toxin | Unknown |
| 780 | Scombrotoxin/Octopus (suspect)/CA/May 2018 | 5/22/2018 | Seafood Toxin | Unknown |
| 800 | Scombrotoxin/Albacore Tuna (suspect)/VT/Jul 2018 | 7/27/2018 | Seafood Toxin | Unknown |
| 803 | Ciguatoxin/Jack (suspect)/ OH/Aug 2018 | 8/3/2018 | Seafood Toxin | Unknown |
| 822 | Ciguatoxin/Hogfish/PR/Sep 2018 | 9/28/2018 | Seafood Toxin | Unknown |
| 821 | Scombrotoxin/Tuna (suspect)/OH/Sep 2018 | 9/28/2018 | Seafood Toxin | Unknown |
| 831 | Ciguatoxin/Barracuda/PR/Oct 2018 | 10/29/2018 | Seafood Toxin | Unknown |
| 853 | Ciguatoxin/Grouper/HI/Jan 2019 | 1/30/2019 | Seafood Toxin | Unknown |
| 854 | Ciguatoxin/Jackfish/HI/Jan 2019 | 1/31/2019 | Seafood Toxin | Unknown |
| 855 | Ciguatoxin/Blue Runner or Horse-eye Jack/FL/Feb 2019 | 2/4/2019 | Seafood Toxin | Unknown |
| 868 | Scombrotoxin/Mahi Mahi/OH/Apr 2019 | 4/1/2019 | Seafood Toxin | Unknown |
| 904 | Scombrotoxin/Tuna/Sep 2019 | 9/4/2019 | Seafood Toxin | 50 |
| 934 | Adverse Reaction/Energy Drink (suspect)/IL/Dec 2019 | 12/16/2019 | Unknown | Unknown |
| 999 | Acute Liver Failure/Alkaline Water (suspect)/Mar 2021 | 3/13/2021 | Industrial Chemical | 20 |
| 1060 | Adverse Illness Events/Keto Shakes (suspect)/Mar 2022 | 3/22/2022 | Unknown | 5 |
| 1064 | Adverse Illness Events/Cereal (suspect)/Apr 2022 | 4/4/2022 | Unknown | 120 |
| 1076 | Adverse Illness Events/Lentil and Leek Crumbles/Jun 2022 | 6/21/2022 | Natural Toxin | 362 |
| 1152 | Adverse Illness Events/Morel Mushroom/MT/Apr 2023 | 4/21/2023 | Natural Toxin | 51 |
| 1198 | Lead and Chromium/Fruit Puree Pouches/Nov 2023 | 11/1/2023 | Toxic Element | 87 |
| 1233 | Adverse Illness Events/Mushroom Microdosing Products/Jun 2024 | 6/4/2024 | Pharmaceutical or Illicit Drug | 169 |
| 1264 | Adverse Illness Event/Saag Dishes/Sep 2024 | 9/13/2024 | Natural Toxin | 26 |
